# Testicular seminoma – unusual histology and staging with sub epithelial spread of seminoma along the vas deferans

**DOI:** 10.1186/1471-2490-6-5

**Published:** 2006-03-01

**Authors:** Chris J Lockett, Ghulam M Nandwani, Simon R Stubington

**Affiliations:** 1 Department of Urology, Michael Heal Unit, Leighton Hospital, Middlewich Road, Crewe, Cheshire, CW1 4QJ, UK

## Abstract

**Background:**

The route of local and metastatic spread of testicular seminoma is well recognised and accepted. The spread is via lymphatics to the paraaortic nodes.

**Case Presentation:**

We present a case report of testicular seminoma in a 56 year old man with previously unreported histological findings. In this case seminoma tumour cells did not appear to have spread by the expected lymphatic route. There was no involvement of retro-peritoneal para-aortic lymph nodes. The tumour appeared to have spread directly along the vas deferans in the sub epithelial plane to the mesenteric lymph nodes.

**Conclusion:**

This type of seminoma tumour spread has not previously been described and it is not a recognised route for metastasis by seminoma tumour. In this case the macroscopic clinical appearance was of a stage I tumour with normal tumour markers. However, the pathological stage of the tumour was surprisingly increased to stage III on the basis of histology and CT radiological findings. We present the unusual histological findings. In view of this unusual histological finding we reinforce the need for accurate staging and for resection of the spermatic cord close to the deep inguinal ring. Accurate staging is crucial in planning the treatment and follow up of seminoma and determines the prognosis.

## Background

We present a case report of testicular seminoma in a 56 year old man where the histological findings were unusual and the route of spread has not previously been reported. Accurate staging is crucial in planning the treatment and follow up of seminoma and determines the prognosis. The American Joint Committee on Cancer (AJCC) tumour node metastasis (TNM) staging system is widely used to stage testicular tumours and includes tumour markers in the staging system. Stage I disease is subdivided according to the T stage and Tumour marker levels. Stage II disease is subdivided according to the volume of retroperitoneal lymph node involvement and stage III disease according to the degree of metastatic involvement and serum tumour markers [1]. In this case the clinical stage appeared to be stage I, but was surprisingly upstaged to stage III by histological findings in the vas deferans and CT radiological findings.

## Case presentation

A 56 year old male presented with a non tender hard atrophic left testicle. The rest of the examination was normal. There had been no previous scrotal surgery or trauma.

**Figure 1:**
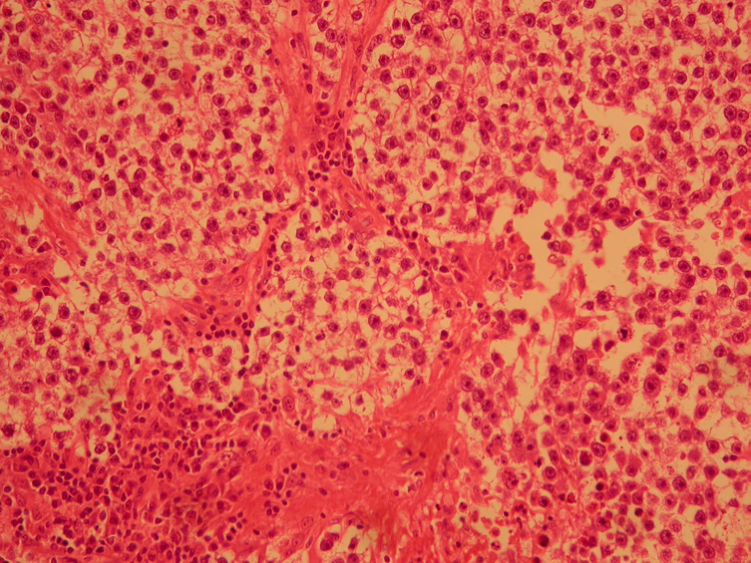
Histological appearnce of the left testis demonstrating testicular seminoma.

**Figure 2:**
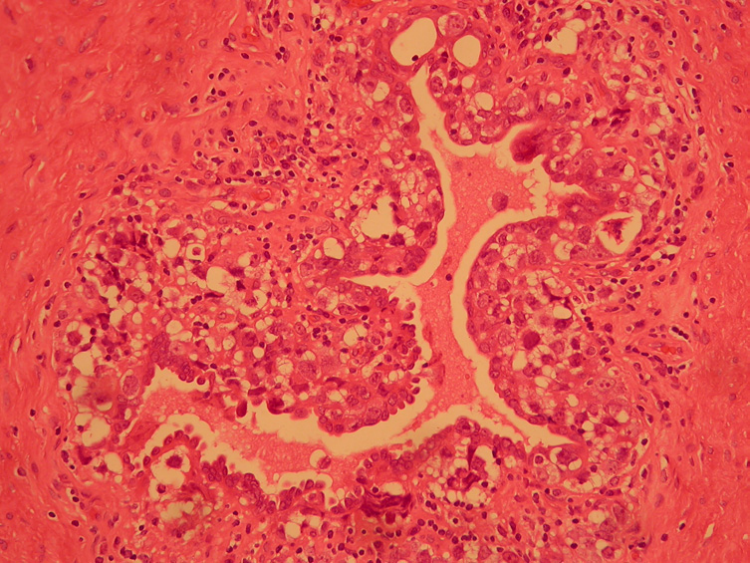
Histological appearance of left vas deferans with sub epithelial seminoma cells.

Tumour markers including lactate dehydrogenase (LDH), human chorionic gonadotrophin (HCG), alpha fetoprotein (AFP) and prostate specific antigen (PSA) were normal. His total white cell count was normal. An ultrasound of the scrotum demonstrated a localised tumour of the testis. Chest x-ray was normal. A standard left radical orchidectomy was performed by the inguinal approach. The tumour appeared to be confined to the upper pole of the testis and was clinically described as T1 or T2. Macroscopic histological examination suggested no involvement of the tunica albuginea, epididymis or spermatic cord. Microscopic examination demonstrated infiltration of the testis by uniform ovoid cells with clear cytoplasm, vesicular nuclei and prominent nucleoli consistent with seminoma (Fig 1). Tumour involved the tunica albuginea but did not extend through it. There was no vascular invasion. Surprisingly, seminoma cells were present beneath the epithelial lining of the vas deferans extending to the spermatic cord resection margin but nowhere else within the spermatic cord. (Fig 2). The microscopic slides gave the appearance of sub epithelial spread of seminoma cells along the vas deferans. Other structures in the spermatic cord were not involved.

**Figure 4:**
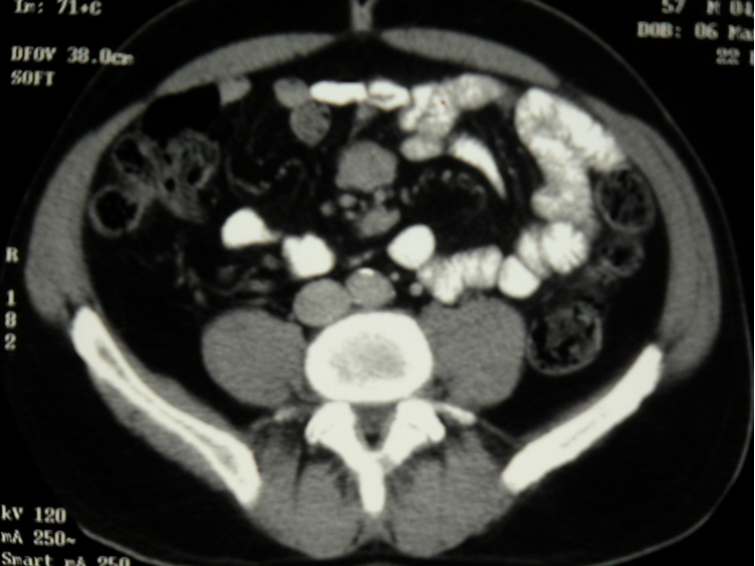
CT scan showing a mesenteric deposit of tumour metastases prior to chemotherapy with no apparent para aortic lymph node enlargement.

**Figure 5:**
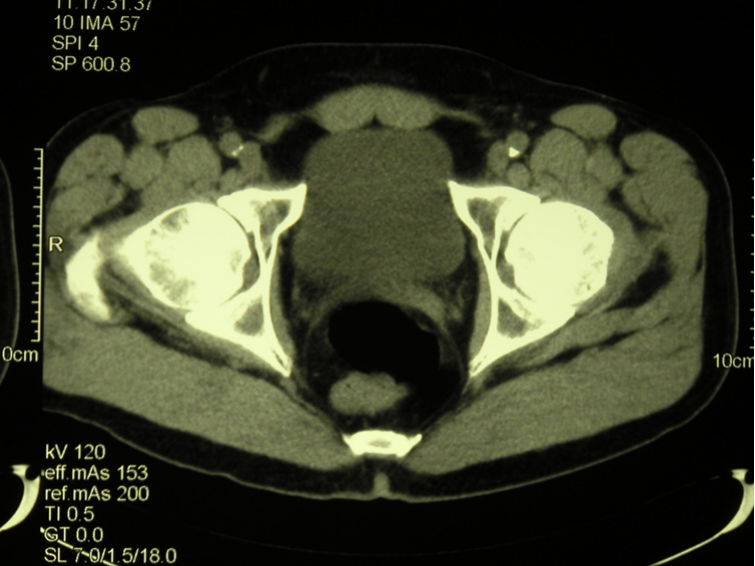
CT scan showing complete regression of pelvic metastases after chemotherapy.

**Figure 6:**
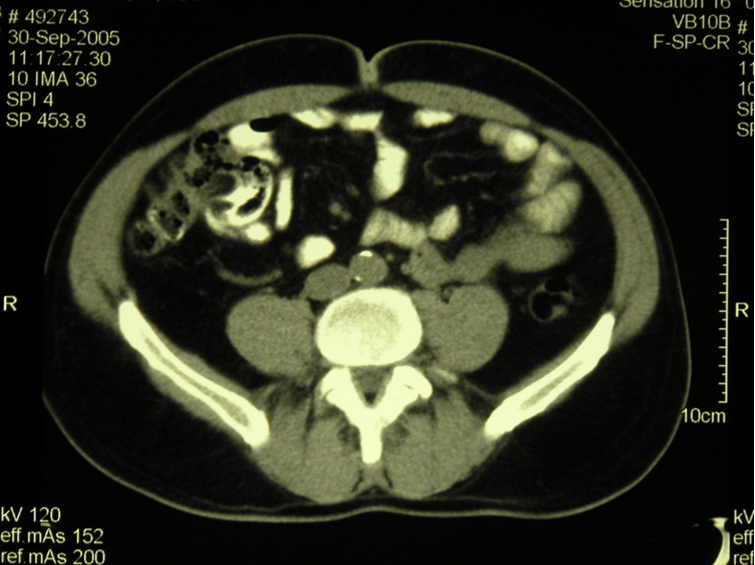
CT scan showing complete regression of mesenteric metastases after chemotherapy.

A post operative CT scan demonstrated two separate masses. A 2.8 cm by 2.3 cm central abdominal mesenteric mass and a 7.6 cm by 5.4 cm irregular heterogeneous lobulated soft tissue mass within the pelvis arising from the prostate and extending into the mesorectal fascia. There were no enlarged retroperitoneal, pelvic, inguinal or thoracic lymph nodes. (Fig 4 and 5). The chest CT scan was normal. The patient has received chemotherapy and is currently under follow up according to standard EUA guidelines. There is no evidence of residual or recurrent disease. The CT scan performed after completion of the course of chemotherapy demonstrated complete resolution of the two metastatic masses. (Fig 5 and 6).

## Conclusion

Accurate staging is important because it dictates the management of the disease. Inaccurate staging has been reduced to approximately 20% in T1 – T3, N0, M0 disease using modern staging techniques [2]. Radiotherapy is the treatment of choice for low stage seminoma (IIB or less). High stage of seminoma (above IIB) can have durable periods of remission with cytotoxic chemotherapy [3]. The histological findings in this case were surprising as the clinical tumour stage was initially believed to be T1 or T2, N0, M0, S0 with an extremely good prognosis. Histological involvement of the spermatic cord, however, upstaged the primary tumour to a T3 tumour with positive resection margins. The main factors for prognosis are described in International Germ Cell Consensus Classification. The prognosis according to this staging system is good. Spermatic cord involvement is normally seen within the main cord vasculature or grossly involving the cord. In this case the seminoma cells appeared to be spreading along the spermatic cord by "creeping" along underneath the epithelial lining of the vas deferans. This mode of spread has not previously been described. Following CT scanning the pathological stage was T3, N0, M1 b, S0. This is likely to be a stage IIIc testicular cancer, but does not fit "neatly" within the staging criteria described in the 1997 AJCC staging system. Seminoma spreads most commonly by the lymphatic route alone. The lymphatics that accompany the testicular vessels exit from the testis through the inguinal ring to the retroperitoneal para-aortic lymph nodes and typical patterns of spread occur according to the side of the primary tumour are well recognised [4]. In a study of the microvasculataure of the rat vas deferens by Ohtani and Gannon, [5] the arterial supply and venous drainage has been described in great detail. In rats a sub epithelial capillary network has been identified. It is possible that this capillary network exists in humans. In this case the seminoma may have spread along a similar sub epithelial capillary network. Tumour spread along a speculated sub capillary network has certainly not been previously reported in man. The recognised route of spread of testicular seminoma is via the lymphatics to the para aortic nodes. In this case the pattern of spread did not appear to follow the normal pathway of lymphatic drainage to the para-aortic nodes. It appears to have spread to the mesentery by a direct route below the epithelium of the spermatic cord. This route of metatsatic spread of seminoma has not previously been described in man.

Testicular cancer is curable in the majority of cases. The main factors determining the prognosis are the stage, (including tumour markers), early orchidectomy and early chemotherapy. Patients who develop a germ cell tumour before the age of 50 years have better 10 year relative survival (91%) than those who develop disease after the age of 50 years (84%) [6]. A pre operative CT scan in this case would have helped to stage the tumour more accurately from the outset but would not have altered the management of this patient. The sequence of treatment first before CT scanning is widely accepted and this should be followed in all cases.

The prognosis and period of remission remains uncertain in this case. This case reinforces the need for high spermatic cord ligation and excision at the deep inguinal ring and immediate CT staging. This should be performed even in patients who are thought to have low stage disease and when tumour markers are normal. The histological and radiological findings may be quite unexpected.

## Abbreviations

CT – computed tomography, TNM – tumour, node metastais staging system, AJCC – American Joint Committee on Cancer

## Competing interests

The author(s) declare that they have no competing interests.

## Authors' contributions

All three authors have been involved in completing this publication

## Pre-publication history

The pre-publication history for this paper can be accessed here:



## Figures and Tables

**Figure 3:**
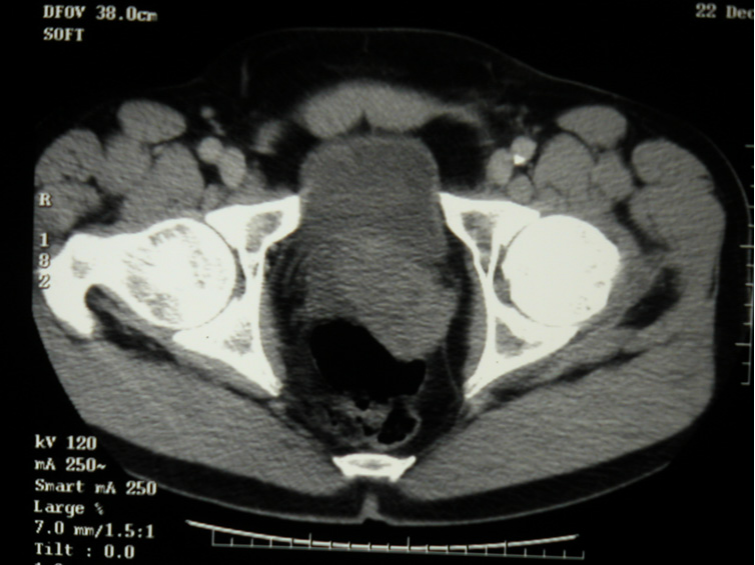
CT scan showing a prostatic deposit of tumour metastases prior to chemotherapy.
